# Is All Human Hearing Cochlear?

**DOI:** 10.1155/2013/147160

**Published:** 2013-12-19

**Authors:** Seyede Faranak Emami

**Affiliations:** Department of Audiology, Faculty of Rehabilitation, Hamadan University of Medical Sciences and Health Services, Hamadan, Iran

## Abstract

The objective of this cross-sectional study was to investigate *the possibility that the saccule may contribute to human hearing*. The forty participants included twenty healthy people and twenty other subjects selected from patients who presented with benign paroxysmal positional vertigo to Audiology Department of Hazrat Rasoul Akram hospital (Tehran, Iran). Assessments comprised of audiological evaluations, cervical vestibular evoked myogenic potentials (cVEMPs), recognition of spoken phonemes in white noise (Rsp in wn), and auditory brainstem response to 500 Hz tone burst (ABR_500 HZ_). Twenty affected ears with decreased vestibular excitability as detected by abnormal cVEMPs revealed decreased scores of Rsp in wn and abnormal findings of ABR_500 HZ_. Both unaffected and normal ears had normal results. Multiple comparisons of mean values of cVEMPs and ABR_500 HZ_ between three groups were significant (*P* < 0.05, ANOVA). The correlation between RSP in wn and p13 latencies was significant. The peak-to-peak amplitudes showed significant correlation to RSP in wn. The correlation between RSP in wn and the latencies of n23 was significant. In high-level of noisy competing situations, healthy human saccular sensation can mediate the detection of low frequencies and possibly help in cochlear hearing for frequency and intensity discrimination. So, all human hearing is not cochlear.

## 1. Introduction 

The pattern of hypersensitivity of the vestibular system to sound stimulation consistent with the clinical sign known as *Tullio* phenomenon (the generation of vestibular symptoms during exposure to high-intensity sounds) was first described by Pietro Tullio (1929) [1–3]. Then, Békésy (1935) reported that loud sounds evoked head movements. He argued that these responses were due to acoustic stimulation of the vestibular system as they persisted even after the stimulation induced temporary deafness. The continous observations led researchers to propose humans saccule as the end organ activated by sound [4, 5]. Finally, John Tait (1936) speculated about a possible auditory role for the otolith organs in amniotes (reptiles, birds, and mammals), including humans, but at past there was no direct evidence for that idea [6].

The evidence from birds and mammals, including human, has shown that the saccule, a hearing organ in many lower vertebrates, has retained some of its ancestral acoustic sensitivity [7, 8]. The saccule lies beneath the stapes and is the vestibular end organ most sensitive to sound. Neurons from the saccule that respond to tilts also respond to acoustical stimulation [3]. The acoustic stimulation of the saccule has behavioral significance in mammals [5, 9] and contributes to behavioral responses to low frequencies [6, 10, 11].

Recently, The researcher could measure bioelectrical responses when the mastoid of profoundly deaf subjects who had a normally functioning vestibular apparatus was stimulated with 100 Hz sinusoidal vibration [12, 13]. The data available for hearing impaired subjects show some evidence of changes in the pattern of discriminability for tones above saccular threshold [5]. Compensatory role of saccule in deaf children and adults makes this organ the ending point of the phonetic information (perception) but also the starting point of its regulation (production) [14]. Therefore, the objective of this study was to investigate the possibility that the saccule may contribute to human' hearing.

## 2. Materials and Methods

This cross-sectional comparison study consisted of twenty healthy peoples and twenty subjects with benign paroxysmal positional vertigo. They were consecutive patients, who presented to the audiology department of Hazrat Rasoul Akram hospital (Tehran, Iran) from May 2012 through december 2012. We screened all volunteers of eligible patients with benign paroxysmal positional vertigo (*n* = 67) during seven months (census method for sampling strategy); twenty patients were included and forty-six other patients were excluded. The diagnosis of patients with benign paroxysmal positional vertigo was based on medical history and findings of characteristic nystagmus (torsional up beating nystagmus with latency and fatigue lasting less than 1 min) and subjective vertigo in the Dix-Hallpike test [15]. The study was approved by the Iran university ethics committee.

The *exclusion criteria* consisted of history of ear infections and middle ear diseases, which can interfere with cVEMPs measurements and conditions that can cause abnormal auditory function. This list included history of head trauma, ototoxic drugs, otosclerosis, labyrinthitis, cardiac and metabolic diseases, heart failure, anemia, hypothyroidism, hyperthyroidism, diabetes mellitus, hypertension, and various neurological diseases (vertebrobasillar insufficiency, temporal lobe epilepsy, multiple sclerosis, central nervous system tumors, and cerebellar infarction, among others).

The *inclusion criteria* involved normal function of hearing, middle ear pressure, auditory brainstem-pathway, and abnormal function of saccule (for reducing of unsatisfactory agents and for producing a similar quality distribution of the patients, we only selected the subjects with benign paroxysmal positional vertigo).

A handedness questionnaire was also administered. All the subjects were right-handed, they were native speakers of the Persian language (unilinguistic).

### 2.1. Assessments

Testing was bilateral and consisted of pure tone audiometry, impedance acousticmetry, click-evoked auditory brainstem response, and videonystagmography, which were employed for reviewing of the inclusion criteria. Then, all of participants had normal function of hearing, middle ear, and auditory brainstem-pathway. Also, for evaluating our main variables, we used cVEMPs, Rsp in wn and ABR_500 HZ_. All of the tests were performed on same day and in each step of evaluation, when the procedure was completed for one test, subjects were given a short break, and the whole procedure was repeated for another.

### 2.2. Cervical Vestibular Evoked Myogenic Potentials (cVEMPs)

The cVEMPs results for the normal group were used as normative data. The values for latency and cVEMPs asymmetry ratio were calculated as mean ± two standard deviations. Any cVEMPs asymmetry ratio above the calculated upper limit was considered to reflect depressed response on the side with lower amplitude findings and interpreted as abnormal. The latencies longer than the calculated upper limit were interpreted as abnormal. Absence of a meaningful waveform with p13 and n23 (no response) was also considered as an abnormal finding [3, 5].

### 2.3. II-Auditory Brainstem Response to 500 HZ Tone Burst (ABR_500_ ****_HZ_)

The evaluation of ABR_500 HZ_ was done to study low-frequency sensitivity. The patients were placed in the supine position on a gurney within a sound-treated room. Noninverting electrode was placed at the high forehead and inverting electrode on ipsilateral mastoid and ground electrode on contra lateral. Electrode impedances were roughly equivalent and were <5 kilohms at the start of the test. Responses to 2000 stimuli were averaged, and each response (rate of 31.7/s) was replicated. Responses were filtered from 30 to 3000 Hz. The stimulus in our paradigm was a 2-0-2 tone burst (500 Hz, 120 dB_SPL_; noise = 90 dB_SPL_), Blackman-windowed. A response window of 25 ms was used when responses were recorded for all tone burst stimuli [16, 17]. The ABR_500 HZ_ was abnormal, when wave V not found or exceeded the normal limits of our laboratory.

### 2.4. Recognition of Spoken Phonemes in White Noise (Rsp in wn)

The evaluation of Rsp in wn was done to study low-frequency sensitivity [18, 19]. Regarding phonological properties of the Persian language, we combined the vowel /*e*/ with voiced consonants (/*m*/,  /*n*/,  /*h*/,  /*b*/,  /*k*/,  /*p*/,  /*r*/,  and  /*l*/) and created two homogeneous monosyllabic phoneme consonant-vowel (CV) lists, which stimulate low frequency neurons (list-1:  /*me*/,  /*ne*/,  /*he*/,  /*be*/,  /*ke*/,  /*pe*/,  /*re*/,  and  /*le*/;  list-2:  /*re*/,  /*be*/,  /*ne*/,  /*he*/,  /*le*/,  /*me*/,  /*ke*/,  and  /*pe*/). They presented at 10 dB signal to noise ratio (signal = 95 dB_Sl_ and white noise = 85 dB_Sl_) to subjects' ipsilateral test ear, at the same time. We assessed their signals via short-time frequency analysis, which was loaded into the Matlab workspace.

The test was done by one female speaker, who was a native of the Persian language and had not the dialect. She did not know about the case or the control subjects, testing was blinded, and the monitoring of live voice was done. The linguistic and psychologica factors of familiarity, redundancy, and emotional loading were controlled from person to person.

### 2.5. Statistical Analyze

All analysis was done by means of the statistics software SPSS_17_. Data were expressed as mean ± standard deviation and as percentages. Kolmogorov-Smirnov test was used for evaluation of normal test distribution. One-way ANOVA was used to compare findings among the three groups. Tukey's least significant difference (Tukey HSD) test was chosen as the post hoc test. Also, Spearman's rank correlation coefficient (Spearman's rho) calculated the relationship between the groups. *P*-value of  <0.05 was considered to indicate statistical significance.

## 3. Results

### 3.1. Cervical Vestibular Evoked Myogenic Potentials (cVEMPs)

**Figure 1:**
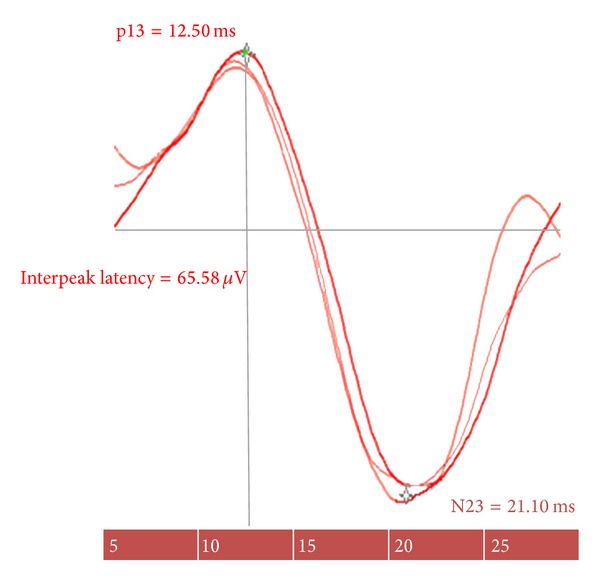
The cervical vestibular evoked myogenic potentials (cVEMPs) in an healthy person.

The mean latencies at p13 and n23 in healthy group (40 normal ears) were 13.37 ± 1.9 ms and 19.56 ± 2.52 ms (Table 1 and Figure 1). The upper limits for them were 17.17 ms and 24.6 ms, respectively. The mean peak-to-peak amplitude was 45.57 ± 28.7 *μ*v, and the upper limits for this ratio were 24.06% (clinical investigations provide evidence that, for adult subjects less than 60 years old, an asymmetry ratio of ≤0.34 or 0.35% is considered normal [20]).

The *affected ears* of the patient group with decreased vestibular excitability as detected by abnormal cVEMPs had decreased amplitudes and delayed latencies in nineteen, and absent responses in one. The mean p13 latencies and n23 latencies were 17.97 ± 1.52 ms and 26.13 ± 1.37 ms, respectively. The mean peak-to-peak amplitude was 35.59 ± 3.34 *μ*v.

### 3.2. Auditory Brainstem Response to 500 HZ Tone Burst (ABR_500_ ****_HZ_)

**Figure 2:**
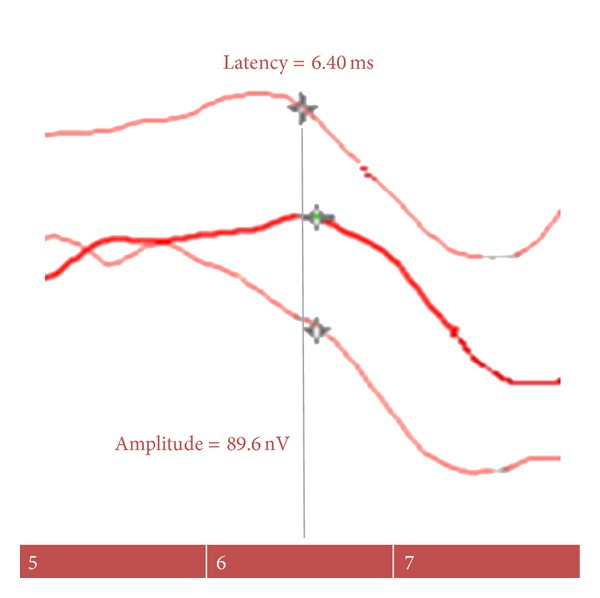
The auditory brainstem response to 500 HZ tone burst (ABR_500 HZ_) in an healthy person.

ABR_500 HZ_ was recordable bilaterally from all healthy persons (Figure 2). The mean amplitudes and the mean latencies values for wave V were 1.09 ± 0.62 and 5.95 ± 0.57, respectively. The wave V had lower amplitudes (the mean = 0.64 ± 0.31) and longer latencies (the mean = 6.56 ± 0.77) in affected ears (Table 2).

### 3.3. II-Recognition of Spoken Phonemes in White Noise (Rsp in wn)

Rsp in wn obtained for the normal group (mean = 96.87 ± 5.53, minimum = 88%, and maximum = 100%). Affected ears had decreased Rsp in wn scores (mean = 60.31 ± 10.84, minimum = 50%, maximum = 88%) (Table 3).

### 3.4. The Main Outcome Measures

Multiple comparisons of mean p13 latencies, mean n23 latencies, and mean peak-to-peak amplitudes of the cVEMPs between three groups were significant (*P* < 0.05, ANOVA test). Comparisons of mean p13 latencies of the cVEMPs in the affected ears versus the unaffected ears and the control group were significant (*P* = 0.00, Tukey HSD). Comparisons of mean n23 latencies of the cVEMPs in the affected ears versus the unaffected ears and the control group were significant (*P* = 0.00, Tukey HSD). Comparisons of mean peak-to-peak amplitude of the cVEMPs in the affected ears versus the unaffected ears and the control group were significant (*P* = 0.02, Tukey HSD). Multiple comparisons of mean wave V latencies and mean wave V amplitudes (*P* < 0.05, ANOVA test) of the ABR_500 HZ_ between three groups were significant. Comparisons of mean wave V latencies of the ABR_500 HZ_ in the affected ears versus the unaffected ears and the control group were significant (*P* = 0.01, Tukey HSD). Comparisons of mean wave V amplitudes of the ABR_500 HZ_ in the affected ears versus the unaffected ears and the control group were significant (*P* = 0.01, Tukey HSD).

The correlation between RSP in wn and p13 latencies was significant (*P* = 0.00,  *r* = −0.30). The peak-to-peak amplitudes showed significant correlation to RSP in wn (*P* = 0.04,  *r* = 0.01). The correlation between RSP in wn and the latencies of n23 was significant (*P* = 0.00,  *r* = − 0.30).

### 3.5. The Main Results

We obtained an association between cVEMPs and Rsp in wn and a relation between cVEMPs and ABR_500 HZ_.

## 4. Discussion 

Twenty affected ears of the patient group with decreased vestibular excitability as detected by abnormal cVEMPs revealed abnormalities in ABR_500 HZ_ and Rsp in wn. Whereas, both healthy and unaffected ears presented normal results. Since, during listening in quite, auditory neurons in primary auditory cortex respond to both best frequency tones (low frequencies) and nonbest frequencies, and through perception in noise, they respond only to best frequencies [18], I concluded saccular stimulation in unaffected ears can contribute to the affective quality of loud low frequencies.

Also, in presence of noise, the loss of the temporal fine structure does not allow listeners to acquire a sufficient harmonic pitch information to segregate the signal and maskers. So, speech perception will be difficult to understand. Whereas, during perception of speech in quiet, envelope signals dominate over temporal fine structure cues. It is important to note that without good access to temporal fine structure, it is difficult to derive the voicing low frequency, a cue that helps formation of auditory objects and segregation of multiple sounds [19, 21]. Then, saccular hearing which stimulates with low frequency is an effective reinforcer for cochlear hearing. It can mediate the detection of low frequencies and cooperates to frequency and intensity discrimination.

However, low frequency cues of the sound spectrum have very important roles in auditory function, which can stimulate the saccular afferents. Low frequencies are always at rates that correspond to fundamental frequencies [19]. Auditory nerve fibres synchronized strongly to low frequencies in the spectra of the vowels [21].

The “phase-locked rate” or “synchronized rate” of the auditory nerve fibers, which is related to low frequencies lies in the frequency range of the saccule [22]. Also, low-frequency sounds, like a single violin note or a syllable in speech, which are in the range of saccular sensation, convey the phonetic and pitch information and prosodic cues, such as intonation and stress [19, 21, 23].

In other hand, profoundly deaf subjects with a normally functioning saccular system might obtain useful information from sound when stimulated adequately [12]. The recent experience demonstrates the phonetic role of saccule in the regulation of the human voice and provides the basis for further development of this topic. The high response of the saccule allows phonemic self-regulation, compensating the low/absent tone-verbal feedback. The specific sensitivity of the saccule in the low frequency range, and its representation in cortical areas suggests the integration of the saccular information in neuronal networks [14].

Therefore, saccule not only responds best to low-frequency high-intensity sound. But also, in clamor conditions may contribute to the hearing of this frequency band. After all, I strongly belive saccule is an ancestor reinforcer for cochlea and all human hearing is not cochlear. The findings observed in adults encourage me to evaluate the role of saccular hearing also in healthy children.

### 4.1. Implications for Clinical Practice

I recommend the cVEMPs evaluation should be done in the battery approach tests of the auditory function for normal populations. In high level noise, it can predict humansignal detection abilities; these skills are affective in hearing and perception-production functions. But, the evaluation of them in healthy adults is mostly unseen.

## Figures and Tables

**Table 1:** The mean of the right and left p13-n23 latencies and amplitudes of cervical vestibular evoked myogenic potentials (cVEMPs) in the patient and the normal groups.

Variable	Normal control ears	Affected ears	Unaffected ears
p13 (ms)	13.37 ± 1.9	17.97 ± 1.52	13.7 ± 0.9
n23 (ms)	19.56 ± 2.5	26.13 ± 1.37	20.16 ± 1.2
Peak-to-peak amplitude (*μ*v)	45.57 ± 28.7	35.59 ± 3.34	43.40 ± 22.6

**Table 2:** The mean of the right and left latencies and amplitudes of auditory brainstem response to 500 HZ tone burst (ABR_500 HZ_) in the patient and the normal groups.

Variable	Normal ears	Affected ears	Unaffected ears
Peak to peak amplitude (*μ*v)	1.09 ± 0.62	0.64 ± 0.31	0.8 ± 0.29
Latency (ms)	5.95 ± 0.57	6.56 ± 0.77	5.64 ± 0.82

**Table 3:** The mean of recognition of spoken phonemes in white noise (Rsp in wn) in the patient and the normal groups.

Subject	Normal ears	Affected ears	Unaffected ears
Rsp in wn	96.87 ± 5.53	60.31 ± 10.84	96.24 ± 2.4

## Abbreviations

cVEMPs: Cervical vestibular evoked myogenic potentials
Rsp in wn: Recognition of spoken phonemes in white noise
ABR_500 HZ_: Auditory brainstem response to 500 HZ tone burst.
